# Development of Small Interfering RNA Loaded Cationic Lipid Nanoparticles for the Treatment of Liver Cancer with Elevated α-Fetoprotein Expression

**DOI:** 10.1021/acsbiomedchemau.4c00061

**Published:** 2024-12-11

**Authors:** Kongpop Duangchan, Nathachit Limjunyawong, Kamonlatth Rodponthukwaji, Teeranai Ittiudomrak, Mattika Thaweesuvannasak, Natsuda Kunwong, Chanatip Metheetrairut, Vorapan Sirivatanauksorn, Yongyut Sirivatanauksorn, Prawat Kositamongkol, Prawej Mahawithitwong, Chutwichai Tovikkai, Kytai T. Nguyen, Chatchawan Srisawat, Primana Punnakitikashem

**Affiliations:** † Department of Biochemistry, Faculty of Medicine Siriraj Hospital, Mahidol University, Bangkok 10700, Thailand; ‡ Research Department, Faculty of Medicine Siriraj Hospital, Mahidol University, Bangkok 10700, Thailand; § Center of Research Excellence in Allergy and Immunology, Faculty of Medicine Siriraj Hospital, Mahidol University, Bangkok 10700, Thailand; ∥ Siriraj Center of Research Excellence in Theranostic Nanomedicine, Faculty of Medicine Siriraj Hospital, Mahidol University, Bangkok 10700, Thailand; ⊥ Department of Surgery, Faculty of Medicine Siriraj Hospital, Mahidol University, Bangkok 10700, Thailand; # Department of Bioengineering, University of Texas at Arlington, Arlington, Texas76019, United States

**Keywords:** liver cancer, alpha-fetoprotein, small interfering RNA, cationic lipid nanoparticles, apoptosis

## Abstract

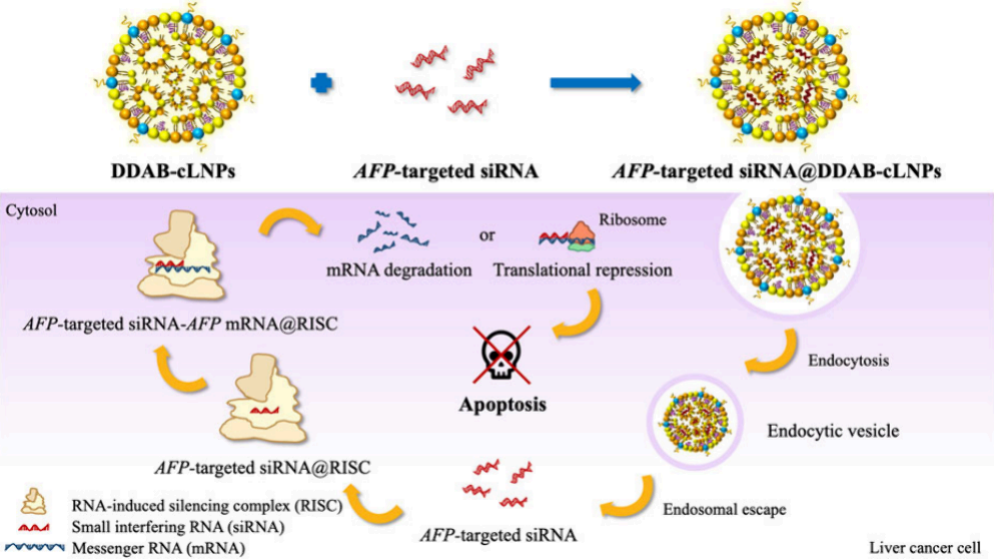

α-Fetoprotein (AFP) is an oncogenic glycoprotein that is overexpressed in most patients with liver cancer. Moreover, it significantly affects tumorigenesis and progression, particularly by inhibiting programmed cell death or apoptosis. The treatment of liver cancer with chemotherapy is currently still in use, but its toxicity is a major concern. Alternatively, targeted therapy, especially small interfering RNA (siRNA)-based therapeutics that utilize siRNA to suppress target gene expression, is a promising cancer treatment approach that can help reduce such drawbacks. However, transporting siRNA into cells is a challenge due to its ease of degradation and limited cell membrane permeability. To overcome this limitation, we fabricated cationic lipid nanoparticles (cLNPs) to deliver *AFP-*targeted siRNA (siAFP) to AFP-producing liver cancer cells. Our results illustrated that these nanoparticles had a high capacity for siRNA encapsulation (>95%) and entered the cancer cells efficiently. Cell internalization of siAFP-loaded cLNPs resulted in the silencing of *AFP* mRNA expression and led to increased apoptotic cell death by inducing caspase-3/7 activity. This suggested that our cLNPs could be used as a powerful siRNA delivery carrier and siAFP-loaded cLNPs might be a useful strategy for treating liver cancer in the future.

## 1 Introduction

Liver cancer is a significant global health concern, ranking as the sixth most common cancer and the third leading cause of death worldwide in 2020. In particular, primary liver cancer (PLC), a malignant neoplasm that originates from any cell of the liver.^1^ Major risk factors for PLC include chronic alcohol consumption, hepatitis B virus (HBV) or hepatitis C virus (HCV) infections, and obesity-related nonalcoholic steatohepatitis (NASH).^2−5^ α-Fetoprotein (AFP) is a glycoprotein typically produced by the liver and yolk sac during fetal development. While serum AFP levels are normally below 20 ng/mL in healthy adults, some patients with liver cancer can exhibit levels exceeding 400 ng/mL.^6,7^ Consequently, AFP is often used as a biomarker for liver cancer diagnosis.^8^

Elevated serum AFP levels in liver cancer patients are associated with disease progression, including the inhibition of apoptosis by suppressing the human antigen R (HuR)-mediated Fas cell surface death receptor (Fas)/Fas-associated death domain (FADD)-mediated extrinsic apoptotic pathway, stimulation of cell proliferation via activating the phosphatidylinositol-4,5-bisphosphate 3-kinase (PI3K)/serine/threonine-protein kinase (AKT)/mammalian target of rapamycin (mTOR) signaling, and promotion of cell invasion and metastasis by upregulating the expression of epithelial cell adhesion molecules (EpCAM), matrix metalloproteinase-2/9 (MMP-2/9), and C-X-C chemokine receptor type 4 (CXCR4).^9−13^ Nowadays, several liver cell lines have been shown to exhibit high levels of *AFP* gene expression, such as SMMC-7721, HuH7, Hep3B, and HepG2. Herein, the HepG2 cell line has been used as a model of AFP-positive cells.^14−16^

Currently, trans-arterial chemoembolization (TACE), the injection of a chemotherapy drug into the blood arteries feeding a malignant tumor, is used to treat some liver cancer patients, for example, HCC patients with intermediate stage or Barcelona clinic liver cancer (BCLC) stage B.^17^ However, systemic chemotherapy encounters challenges such as multidrug resistance and systemic toxicity as it can cause several side effects, including injury and death of surrounding noncancerous cells.^18^ Targeted therapy, using drugs or other substances that directly target cancer cells while minimally affecting normal cells, presents a promising avenue for cancer treatment.^19^ Among targeted therapy approaches, RNA-based therapeutics, including antisense oligonucleotides, synthetic mRNAs, microRNAs, and small interfering RNAs (siRNAs), have garnered attention.^20^

Small interfering RNAs (siRNAs) are synthetic double-stranded RNAs with 21–25 nucleotides,^21^ functioning by silencing target gene expression at the post-transcriptional level through endonucleolytic cleavage of mRNA.^22^ Therefore, they are frequently used to reduce the expression level of undesired genes in cancer cells. However, their therapeutic application is hindered by challenges in their stability and ability for cellular uptake. They are highly susceptible to degradation by exogenous RNases and exhibit poor permeability across the cell membrane due to their hydrophilic nature, negative charge, and relatively high molecular weight.^22−24^ Thus, the development of efficient siRNA carriers is necessary for the success of siRNA delivery into targeted/diseased cells.

Cationic lipid nanoparticles (cLNPs), lipid-based nanoparticles characterized by positive charges on their surface,^25^ have emerged as effective carriers for drug and nucleic acid delivery, especially RNA molecules.^26−28^ A cationic lipid with a quaternary ammonium headgroup named dimethyldioctadecylammonium bromide (DDAB) serves as the main functional group to interact with negatively charged nucleic acids.^29^ These nanoparticles offer enhanced cellular uptake, RNA molecule protection, and high encapsulation efficiency for RNA molecules.^30,31^ In this study, we aimed to develop *AFP*-targeted siRNA-loaded DDAB-cLNPs (siAFP-loaded DDAB-cLNPs) for the treatment of liver cancer with elevated *AFP* expression. We speculated that these cLNPs could improve the efficiency of siRNA delivery to liver cancer cells and that siAFP-loaded DDAB-cLNPs could inhibit cancer progression by silencing *AFP* mRNA expression, thereby inducing apoptosis.

## 2 Experimental Section

### 2.1 Preparation of siRNA

The sense and antisense strands of *AFP*-targeted siRNA (siAFP) and scrambled siRNA (siSCR) were chemically synthesized in modified forms with 2′-fluoropyrimidine. These synthetic siRNAs were obtained from GenePharma Co., Ltd. (Pudong, Shanghai, China). The sequences of siRNA are shown in Table 1. To denature the secondary structure of siRNA, siRNA in the master mix was incubated at 95 °C for 2 min using the T100 Thermal Cycler (Bio-Rad Laboratories, Inc., Hercules, California, USA) and allowed to cool down to room temperature for 20 min in order to anneal sense and antisense strands of siRNA together. The annealed siRNA was kept at −20 °C.

**Table 1:** Sequences of siRNA

Name of siRNA	Type of Modification	Type of Strand	Sequence (5′ → 3′)
Scrambled siRNA	2′-fluoropyrimidine	Sense	GCAGGGUGGCGACCACGUCUU
		Antisense	GACGUGGUCGCCACCCUGCUU
*AFP*-targeted siRNA	2′-fluoropyrimidine	Sense	GCCACUUACAAGGAAGUAAGCAA
		Antisense	GCUUACUUCCUUGUAAGUGGCUU

### 2.2 Preparation of Nanoparticles

Each component, including distearoylphosphatidylcholine (DSPC) (Avanti Polar Lipids, Inc., Alabaster, Alabama, USA), 1,2-dimyristoyl-rac-glycero-3-methoxypolyethylene glycol-2000 (DMG-PEG2000) (Avanti Polar Lipids, Inc., Alabaster, Alabama, USA), cholesterol (FUJIFILM Wako Pure Chemical Corporation, Chuo-Ku, Osaka, Japan), and DDAB (Tokyo Chemical Industry Co., Ltd., Kita-ku, Tokyo, Japan), were dissolved in chloroform. Cationic lipid nanoparticles were fabricated using the thin film hydration method. Briefly, each component was mixed and dried with nitrogen flushing in order to generate a thin film of lipids. Then, this film was hydrated with Tris-HCl solution, pH 7.4, (Bio Basic Inc., Markham, Ontario, Canada) containing annealed siRNA at Nitrogen to Phosphorus (N:P) ratio of 1:16. This was followed by vortexing and sonication at 30% amplitudes with a pulse on 30 s/pulse off 10 s on ice for 10 min. The siRNA-loaded DDAB-cLNPs were stored at 4 °C before further study. For unloaded DDAB-cLNPs, the same protocol was applied without the addition of siRNA solution.

### 2.3 Characterization of Nanoparticles

The morphology of nanoparticles was visualized under a transmission electron microscope (TEM) using a voltage of 100 kV. Briefly, 10 μL of either unloaded or siRNA-loaded DDAB-cLNPs were dropped onto a 200-mesh Formvar/Carbon supported copper grid (MilliporeSigma, Burlington, Massachusetts, USA) and air-dried for 10 min at room temperature. Then, the samples were stained with 10 μL of uranyl acetate (Electron Microscopy Sciences, Hatfield, Pennsylvania, USA) and incubated for 5 min at room temperature. After that, uranyl acetate was wiped from the grid, and then the grid was rinsed with sterile Milli-Q water and air-dried overnight at room temperature. The hydrodynamic size, polydispersity index (PDI), and zeta potential of the nanoparticles were measured using dynamic light scattering (DLS) with the Zetasizer Ultra instrument (Malvern Instruments, Ltd., Spectris plc, Malvern, Worcestershire, United Kingdom).

### 2.4 Encapsulation Efficiency of Nanoparticles

#### 2.4.1 Qualitative siRNA Encapsulation Efficiency of Nanoparticles

The efficacy of siRNA encapsulation was verified by using polyacrylamide gel electrophoresis (PAGE). First, the structure of DDAB-cLNPs was destroyed by using 1% (v/v) Triton X-100 in order to release all the siRNA entrapped in nanoparticles. Then, the sample (siRNA-loaded DDAB-cLNPs with Triton X-100) was mixed with GelPilot DNA Loading Dye (QIAGEN, Hilden, Germany) and loaded into a 20% (w/v) polyacrylamide gel containing Tris-borate-EDTA buffer (Bio-Rad Laboratories, Inc., Hercules, California, USA). Free siRNA without Triton X-100, free siRNA with Triton X-100, unloaded DDAB-cLNPs with Triton X-100, and siRNA-loaded DDAB-cLNPs without Triton X-100 were also run as control groups, and the 25-bp DNA ladder (Thermo Fisher Scientific Inc., Waltham, Massachusetts, USA) was used as a marker to estimate the size of siRNA. This was followed by electrophoresis at 100 V for 3 h. After that, the gel was stained with 0.625 mg/mL ethidium bromide solution (Bio-Rad Laboratories, Inc., Hercules, California, USA), and siRNA bands were visualized using the Azure 300 Chemiluminescent Imager (Azure Biosystems, Dublin, California, USA).

#### 2.4.2 Quantitative siRNA Encapsulation Efficiency of Nanoparticles

The siRNA encapsulation efficiency of DDAB-cLNPs was further confirmed by the Quant-iT Ribogreen RNA Assay Kit (Invitrogen, Thermo Fisher Scientific Inc., Waltham, Massachusetts, USA). Briefly, 100 μL of diluted Ribogreen reagent in RNase-free 1X Tris-EDTA buffer was added to each well of 96-well plates containing siRNA-loaded DDAB-cLNPs samples, either treated or untreated with 1% (v/v) Triton X-100 (MilliporeSigma, Burlington, Massachusetts, USA). Subsequently, samples were incubated in the dark for 5 min. The amount of siRNA was quantified using a microplate reader, with excitation and emission wavelengths set at 485/20 nm and 530/25 nm, respectively. After that, the fluorescence intensity values were used to calculate the percentage of encapsulation efficiency (% EE) as follows:
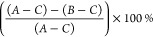
where *A* = fluorescence intensity values of siRNA-loaded DDAB-cLNPs with Triton X-100 (representing total siRNA), *B* = fluorescence intensity values of siRNA-loaded DDAB-cLNPs without Triton X-100 (representing total free siRNA), and *C* = fluorescence intensity values of unloaded DDAB-cLNPs, serving as a blank.

### 2.5 *In Vitro* siRNA Release from Nanoparticles

The release kinetics of siRNA from DDAB-cLNPs was determined by dialysis and spectrofluorometry methods. Briefly, dialysis membrane Spectra/Por Biotech CE MWCO 20,000 (Thermo Fisher Scientific Inc., Waltham, Massachusetts, USA) containing siRNA-loaded DDAB-cLNPs in 40 mM Tris-HCl buffer, pH 7.4, was put in a conical tube containing 10 mL of RNase-free 1X PBS, pH 7.4, and incubated at 37 °C. Then, the amount of siRNA released into the permeate at different time points was quantified using the Quant-iT Ribogreen RNA Assay Kit and analyzed using a microplate reader at 485/20 nm and 530/25 nm excitation and emission wavelengths.^32,33^

### 2.6 Cell Culture

HepG2 cells, AFP-positive human liver cancer cells, were purchased from the European collection of authenticated cell cultures (MilliporeSigma, Burlington, Massachusetts, USA). In this experiment, HepG2 cells were cultured in Dulbecco’s Modified Eagle Medium containing 10% (v/v) fetal bovine serum, and 1% (v/v) Penicillin-Streptomycin (10,000 U/mL) at 37 °C in a 5% CO_2_ incubator.

### 2.7 Cell Transfection

siSCR and siAFP were introduced into the HepG2 cells using the Lipofectamine 2000 Transfection Reagent (Invitrogen, Thermo Fisher Scientific Inc., Waltham, Massachusetts, USA) for 24 h according to the manufacturer’s instructions. After that, the transfection medium was replaced with a fresh cell culture medium before performing other experiments.

### 2.8 Cellular Uptake of siRNA-Loaded Nanoparticles

#### 2.8.1 Qualitative Cellular Uptake of siRNA-Loaded Nanoparticles

In this study, BLOCK-iT Alexa Fluor Red Fluorescent Oligo (BLOCK-iT), serving as a surrogate for siRNA, was purchased from Invitrogen (Thermo Fisher Scientific Inc., Waltham, Massachusetts, USA). HepG2 cells were seeded at a density of 30,000 cells/well in 48-well plates and cultured at 37 °C in a 5% CO_2_ incubator. Then, BLOCK-iT-loaded DDAB-cLNPs were introduced into the cells and incubated for 4 h. Following incubation, the treated cells were washed once with 1X PBS, fixed with 4% (w/v) paraformaldehyde at room temperature for 10 min, and washed twice. To visualize the nucleus, the samples were then stained with 100 nM of 4′,6-diamidino-2-phenylindole (DAPI) for 5 min and washed twice. Finally, 1X PBS was added to the cells to prevent them from drying out during observation under an inverted fluorescence microscope.

#### 2.8.2 Quantitative Cellular Uptake of siRNA-Loaded Nanoparticles

HepG2 cells were seeded at a density of 100,000 cells/well in 24-well plates and incubated at 37 °C in a 5% CO_2_ incubator for 24 h. Subsequently, cells were treated with BLOCK-iT-loaded DDAB cLNPs for 4 h. After that, the treated cells were washed once with 1X PBS and then trypsinized with a 0.25% trypsin-EDTA solution for 3 min. The activity of trypsin was then inhibited with a trypsin-neutralizing solution. Next, the cells were collected into a FACS tube containing FACS buffer (1 mM EDTA and 5% (v/v) FBS in 1X PBS) and centrifuged at 1,500 rpm for 3 min. The supernatant was discarded and the cell pellets were resuspended in FACS buffer. Finally, the uptake of BLOCK-iT-loaded DDAB-cLNPs by HepG2 cells was quantitatively assessed using a flow cytometer.

### 2.9 RNA Extraction and Gene Silencing Evaluation

*AFP* mRNA expression was determined by a quantitative reverse transcription polymerase chain reaction (qRT-PCR). Cells were seeded at a density of 50,000 cells/well in 24-well plates and incubated at 37 °C in a 5% CO_2_ incubator for 24 h. Then, the cells were treated with nanoparticles for 48 h. Following this, RNA from HepG2 cells was extracted using the RNeasy Mini Kit (QIAGEN, Hilden, Germany) according to the manufacturer’s instructions. The extracted RNA was then converted to cDNA using the SuperScript III First-Strand Synthesis System (Invitrogen, Thermo Fisher Scientific Inc., Waltham, Massachusetts, USA) and the reaction was run in the T100 Thermal Cycler (Bio-Rad Laboratories, Inc., Hercules, California, USA). Next, qPCR was performed using KAPA SYBR FAST qPCR Kits (Kapa Biosystems (Pty.) Ltd., Salt River, Cape Town, South Africa) with gene-specific primers as listed in Table 2 on the CFX96 Touch Real-Time PCR Detection System (Bio-Rad Laboratories, Inc., Hercules, California, USA). The reaction cycles were started with initial denaturation at 95 °C for 10 min, followed by 40 cycles of denaturation at 94 °C for 30 s, primer annealing at 57 °C for 30 s, primer extension at 72 °C for 30 s, and a final extension step at 72 °C for 10 min. The relative expression of *AFP* mRNA was calculated using the 2^–ΔΔCt^ method, with the *GAPDH* gene serving as an internal control.

**Table 2:** Sequences of Gene-Specific Primers for qRT-PCR

Gene Name	Type of Primer	Sequence (5′ → 3′)
*GAPDH*	Forward	CCATCTTCCAGGAGCGAGAC
	Reverse	ATGACCCTTTTGGCTCCACC
*AFP*	Forward	CCTTCCTGTATGCACCTACAAT
	Reverse	AACTGTTGCTGCCTTTGTTTG

### 2.10 Cytotoxicity and Caspase-3/7 Activity Measurements

The cytotoxicity of unloaded and siRNA-loaded DDAB-cLNPs on HepG2 cells was evaluated using the CellTiter-Blue Cell Viability Assay (Promega Corporation, Madison, Wisconsin, USA). Briefly, cells were seeded at a density of 10,000 cells/well in 96-well plates and incubated at 37 °C in a 5% CO_2_ incubator for 24 h. Then, the cells were treated with nanoparticles for 48 h. After that, the treated cells were added with the CellTiter-Blue Reagent and incubated at 37 °C in a 5% CO_2_ incubator for 2 h. Next, the plates containing treated cells were shaken for 5 s prior to the measurement of fluorescence intensity at an excitation wavelength of 545/20 nm and an emission wavelength of 590/20 nm using the Synergy HT Multi-Detection Microplate Reader (BioTek Instruments, Inc., Winooski, Vermont, USA). After that, the activity of caspase-3/7 enzymes was determined using the Caspase-Glo 3/7 Assay (Promega Corporation, Madison, Wisconsin, USA). In brief, the Caspase-Glo 3/7 reagent was added to the 96-well plates containing treated cells with the CellTiter-Blue Reagent at a ratio of 1:1 and mixed gently. Then, the reagents were incubated at room temperature in the dark for 1 h. The luminescent signal was measured using the Synergy H1 Hybrid Multi-Mode Microplate Reader (BioTek Instruments, Inc., Winooski, Vermont, USA). Finally, the caspase-3 and 7 activity of each experimental group was normalized to its own cell viability in order to demonstrate the activity of the enzyme in living cells only.

### 2.11 Statistical Analysis

All data were analyzed using GraphPad Prism (GraphPad Software, Inc., San Diego, California, USA) and presented as the mean ± standard deviation (S.D.) of three independent experiments (n = 3). Student’s *t* test was used to compare the means between two experimental groups, while the one-way analysis of variance (ANOVA) with Dunnett’s test was used to compare the means across multiple experimental groups. A *p*-value <0.05 was considered statistically significant.

## 3 Results and Discussion

### 3.1 Physicochemical Characterization of siRNA-Loaded DDAB-cLNPs

**Figure 1:**
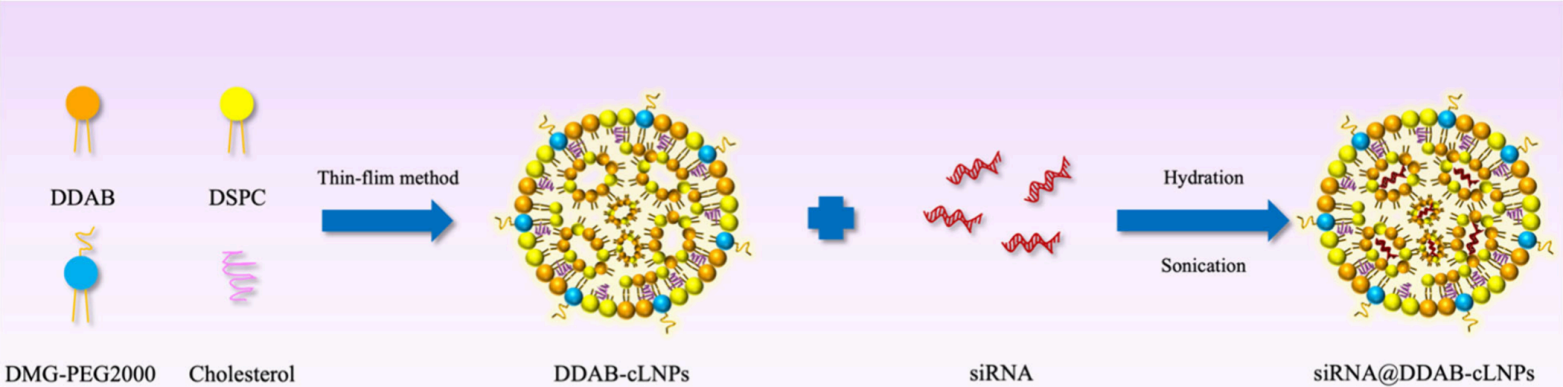
Schematic diagram of siRNA-loaded DDAB-cLNPs. First, all lipid ingredients were combined to fabricate the thin film of lipids by using the thin film method. Then, the thin film was hydrated with a buffer solution containing siRNA molecules and sonicated to incorporate siRNA into nanoparticles.

The thin film hydration method was used to produce DDAB-cLNPs. In order to incorporate siRNA into lipid nanoparticles, a dried thin film of lipids was hydrated with RNase-free Tris-HCl solution at pH 7.4, which stabilized RNA molecules.^34^ The phosphate moiety of siRNA molecules interacted with the quaternary ammonium headgroup of the cationic lipid DDAB (Figure 1).^35^

**Figure 2:**
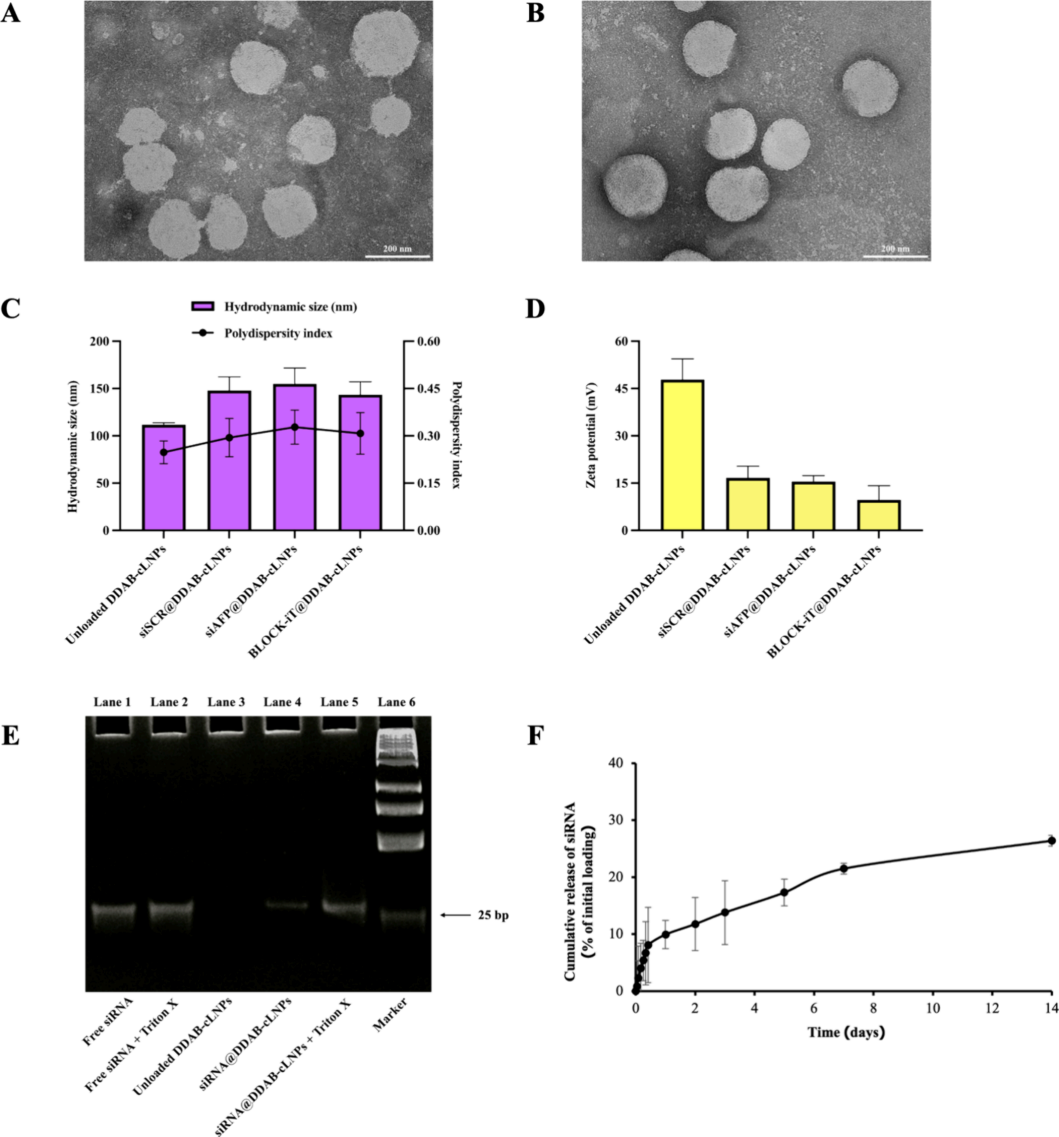
Physicochemical properties of synthesized siRNA-loaded DDAB-cLNPs. (A, B) Morphology of unloaded DDAB-cLNPs and siRNA-loaded DDAB-cLNPs, respectively (scale bar = 200 nm). (C) Hydrodynamic size and polydispersity index of unloaded and siRNA-loaded DDAB-cLNPs. (D) Zeta potential of unloaded and siRNA-loaded DDAB-cLNPs. (E) Qualitative analysis of the siRNA encapsulation efficiency of DDAB-cLNPs. (F) *In vitro* release profile of siRNA from DDAB-cLNPs.

The morphology of unloaded and siRNA-loaded DDAB-cLNPs, visualized by the TEM, confirmed a spherical shape of nanoparticles (Figure 2A,B). The physical properties of nanoparticles, such as hydrodynamic size and zeta potential, were determined using the DLS technique. Unloaded DDAB-cLNPs exhibited a hydrodynamic size of 111.76 ± 2.10 nm and a zeta potential of 47.84 ± 6.61 mV, demonstrating a strongly positive charge (Figure 2C,D). Upon siRNA loading, we found a significant increase in hydrodynamic size, accompanied by an obvious decrease in zeta potential compared to the unloaded DDAB-cLNPs. These observations are consistent with Kedmi et al. (2010), who also observed a decrease in the charge of nanoparticles after encapsulation of siRNA due to its negative charge.^36^ Moreover, particle size distribution, as indicated by PDI values, demonstrated a uniform population for both unloaded and siRNA-loaded DDAB-cLNPs, with PDI values around 0.3 (Figure 2C).^37,38^

Following the assessment of nanoparticle physical properties, the qualitative analysis of siRNA entrapment in DDAB-cLNPs was validated by PAGE. The results exhibited no band in the unloaded DDAB-cLNPs control group and we found that Triton X-100 did not affect the shifting of the siRNA band. In addition, the results demonstrated a prominent siRNA band in lane 5 (from left to right), indicating that most siRNA molecules were encapsulated within the nanoparticles. However, the appearance of faded siRNA in lane 4 (from left to right) suggested the presence of excess siRNA molecules that were not encapsulated into the nanoparticles (Figure 2E). Furthermore, the encapsulation efficiency of siRNA within DDAB-cLNPs was further investigated using the Quant-iT Ribogreen RNA Assay Kit. The results revealed a remarkable siRNA encapsulation capacity exceeding 95%. Our findings are consistent with those of Roces et al. (2020), who reported DDAB-cLNPs capable of encapsulating over 90% of messenger RNA (mRNA).^30^ This study examined the *in vitro* release profile of siRNA from DDAB-cLNPs at pH 7.4 by using dialysis and spectrofluorometry methods. The results showed that approximately 10% of the siRNA was released within 24 h (Figure 2F). This evidence suggests a similar pattern of siRNA release to that reported by Hanafy et al. (2021), who used LNPs containing cationic lipid 1,2-dioleoyl-3-trimethyl ammonium propane chloride (DOTAP) as the carriers of *PD-1*-targeted siRNA.^39^ However, our study employs the nonionizable cationic lipid DDAB, which contains quaternary amine groups that can enhance endosomal rupture via osmotic pressure, thereby facilitating the release of the siRNA payload.^40−42^

### 3.2 Safety Profile of DDAB-cLNPs on Liver Cancer Cells

**Figure 3:**
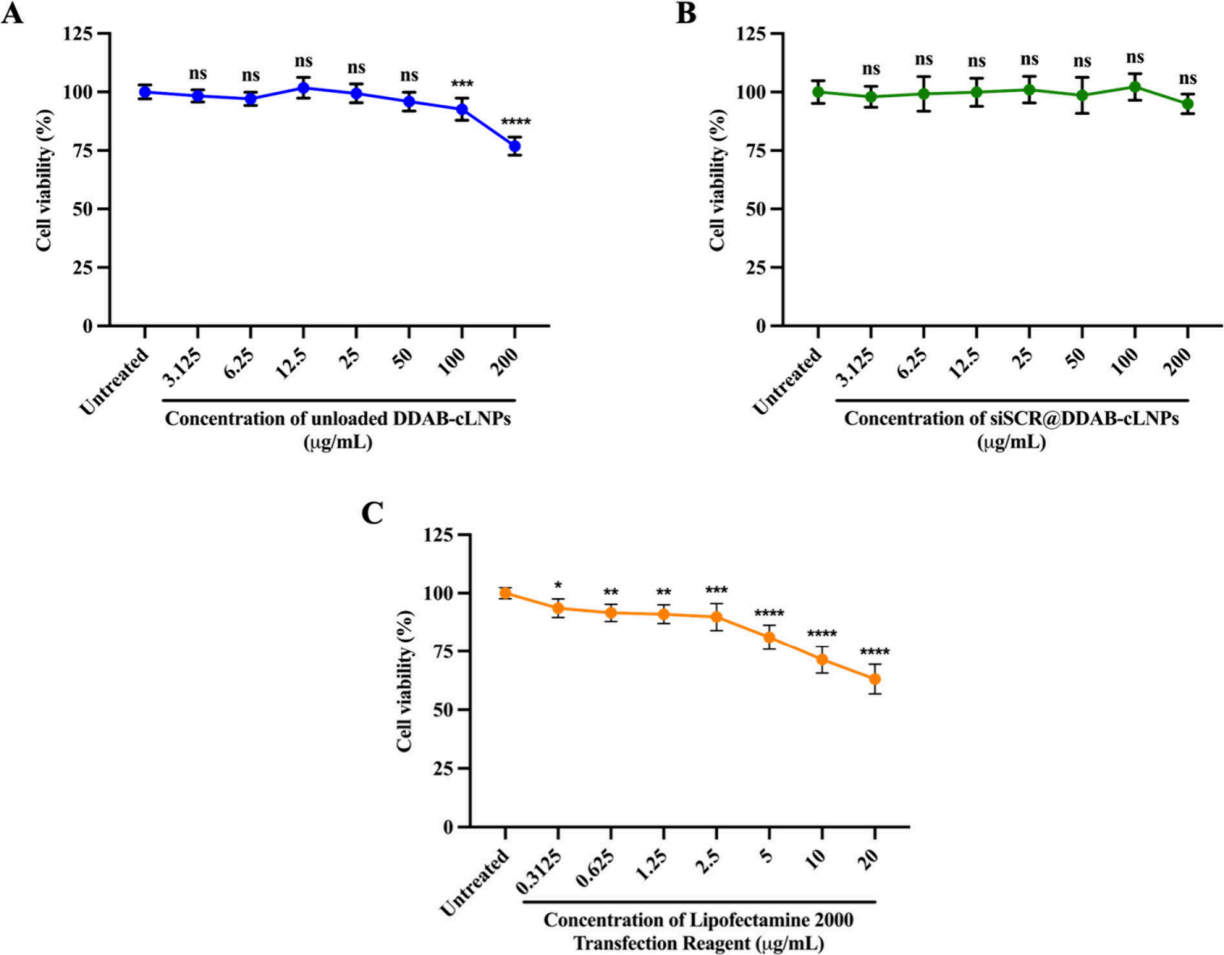
Safety profile of DDAB-cLNPs on HepG2 cell viability. (A) Cytotoxic effects of unloaded DDAB-cLNPs on HepG2 cell viability at 48 h. (B) Cytotoxic effects of siSCR@DDAB-cLNPs on HepG2 cell viability at 48 h. (C) Cytotoxic effects of LP2000 on HepG2 cell viability at 48 h. The data are presented as mean ± SD of three independent experiments. A statistically significant difference between the sample and untreated cell control groups was identified with *p < 0.05, **p < 0.01, ***p < 0.001, and ****p < 0.0001. ns = not significant.

To confirm the cytocompatibility of nanoparticles on HepG2 cells, the impact on cell viability after treating the cells with various concentrations of blank cLNPs was assessed using the CellTiter-Blue Cell Viability Assay. Results at 48 h post-treatment indicated significant toxicity on HepG2 cells treated with unloaded nanoparticles at a concentration of 100 μg/mL (Figure 3A). This finding demonstrated the noticeable toxicity of our nanoparticles to HepG2 cells at high concentrations, possibly due to the strong positive charge from quaternary ammonium molecules in the cationic lipid DDAB.

To further investigate the influence of nanoparticle charge on cellular toxicity, we loaded siSCR molecules into DDAB-cLNPs before treating the cells. The zeta potential was reduced from ≈47 mV to ≈16 mV after siRNA encapsulation (Figure 2D). The results revealed that no significant toxicity of siSCR-loaded DDAB-cLNPs (siSCR@DDAB-cLNPs) was observed across all concentrations (0–200 μg/mL) in HepG2 cells at 48 h (Figure 3B), in contrast to, unloaded DDAB-cLNPs under the same conditions. This suggests that nanoparticle zeta potential or charge indeed plays a crucial role in their impact on cell viability.^43,44^ Moreover, our results corresponded with those of Lechanteur et al. (2018), who reported that the cause of nanoparticle cytotoxicity was mainly from the positive charge of the quaternary ammonium headgroup in cationic lipids.^45^

Additionally, we compared the cytotoxic effects of our synthesized unloaded DDAB-cLNPs to the commercial Lipofectamine 2000 (LP2000). Results illustrated that LP2000 exhibited significant toxicity at 48 h to HepG2 cells even at a low concentration (Figure 3C), highlighting the notably higher cytotoxicity of LP2000 compared to our unloaded nanoparticles.

### 3.3 Cellular Uptake of siRNA-Loaded DDAB-cLNPs

**Figure 4:**
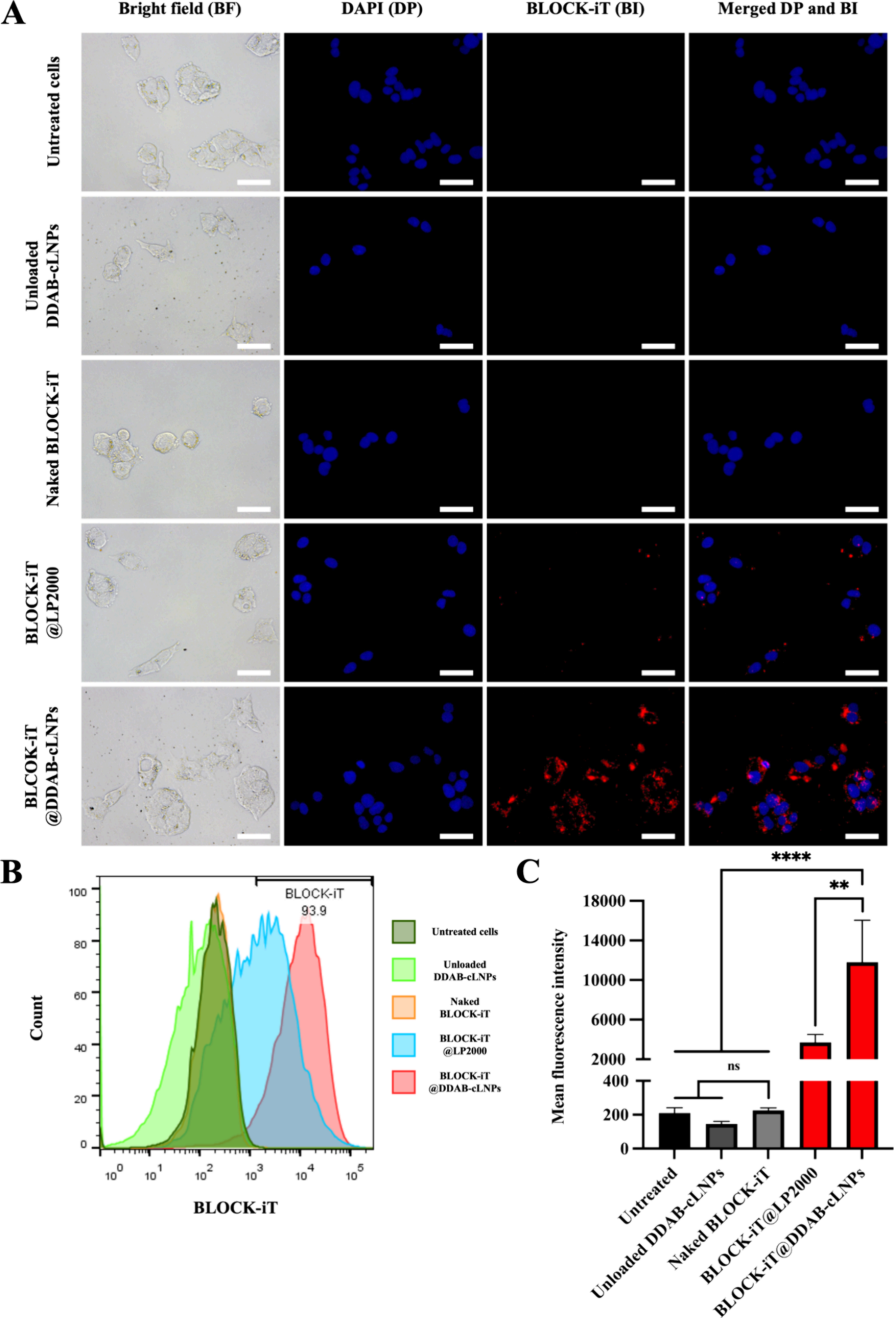
Qualitative and quantitative cellular uptake of siRNA-loaded DDAB-cLNPs in HepG2 cells at 4 h. (A) Fluorescence images of untreated cells, unloaded DDAB-cLNPs, naked BLOCK-iT, BLOCK-iT@LP2000, and BLOCK-iT@DDAB-cLNPs (scale bar = 500 nm). (B) Histogram analysis and (C) MFI analyzed from the flow cytometry technique of HepG2 cells after treatment with BLOCK-iT@DDAB-cLNPs or other control groups. The data are presented as mean ± SD of three independent experiments. A statistically significant difference between the sample and untreated control groups was identified with **p < 0.01 and ****p < 0.0001. ns = not significant.

To investigate the cellular uptake of siRNA-loaded DDAB-cLNPs in HepG2 cells, the BLOCK-iT-loaded DDAB-cLNPs were incubated with HepG2 cells for 4 h, followed by immediate analysis using fluorescence microscopy and flow cytometry techniques. Microscopic images illustrated the intracellular fluorescence signals of BLOCK-iT-loaded DDAB-cLNPs within the treated HepG2 cells. The control groups, such as untreated, unloaded DDAB-cLNPs, and naked BLOCK-iT controls, showed negligible fluorescence signals, demonstrating that siRNA molecules required nanocarriers to deliver them to the cells. This observation aligned with the previous findings showing the challenges of transporting nucleic acids across cell membranes due to their negative charge and hydrophilic properties.^46^ Besides, we also observed a greater fluorescence signal of BLOCK-iT inside HepG2 cells when using DDAB-cLNPs as carriers compared to LP2000 (Figure 4A).

Similarly, flow cytometry analysis showed a significantly increased mean fluorescence intensity (MFI) in HepG2 cells treated with BLOCK-iT-loaded DDAB-cLNPs compared to BLOCK-iT-loaded LP2000 at similar concentrations (Figure 4B,C). In contrast, untreated cells, unloaded nanoparticle-treated cells, and naked BLOCK-iT-treated groups show no fluorescence signal shifts. These results strongly suggest that our nanoparticles could effectively transport siRNA molecules into HepG2 cells.

### 3.4 Determination of *AFP*-Targeted siRNA Knockdown Efficiency

**Figure 5:**
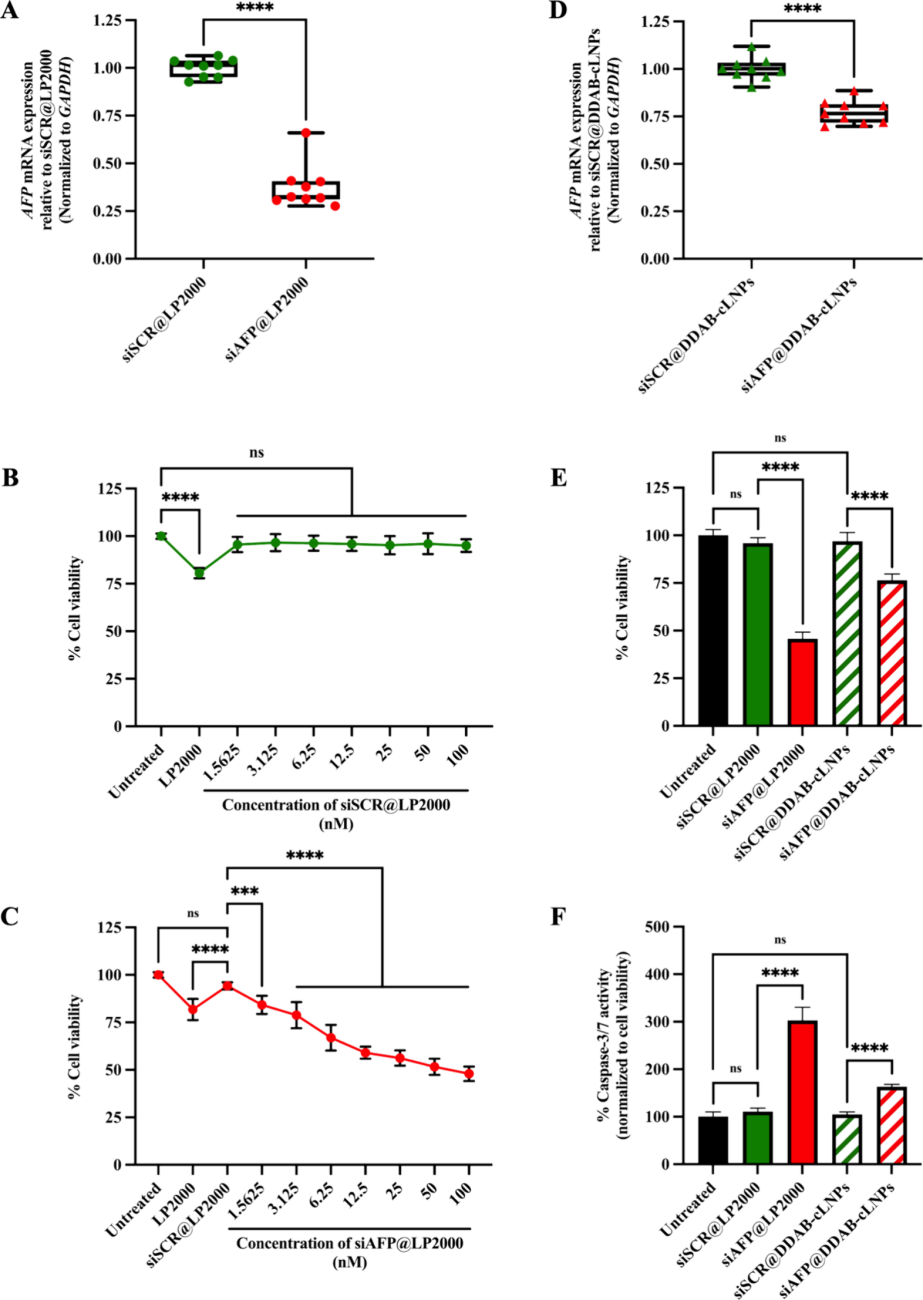
Anticancer effects of siAFP against HepG2 cells using different types of lipid-based nanocarriers. (A) Relative expression level of *AFP* mRNA in HepG2 cells after treatment with siAFP@LP2000 for 48 h. (B) Cytotoxic effects of siSCR@LP2000 on HepG2 cell viability at 48 h. (C) Cytotoxic effects of siAFP@LP2000 on HepG2 cells at 48 h. (D) Relative expression level of *AFP* mRNA in HepG2 cells after treatment with siAFP@DDAB-cLNPs for 48 h. (E) Cytotoxic effects of different types of lipid-based nanocarriers on HepG2 cells at 48 h. (F) Caspase-3/7 activity in HepG2 cells after treatment with different types of lipid-based nanocarriers for 48 h. The data are presented as mean ± SD of three independent experiments. A statistically significant difference between the sample and control groups was identified with ***p < 0.001 and ****p < 0.0001. ns = not significant.

We first assessed the knockdown efficacy of *AFP*-targeted siRNA transfection using LP2000 (siAFP@LP2000) in HepG2. The qRT-PCR analysis demonstrated that at 48 h post-transfection, our siAFP significantly reduced the expression level of *AFP* mRNA in HepG2 cells compared to the scrambled siRNA-treated cells (siSCR@LP2000) control group (Figure 5A). We then further investigated the knockdown efficacy of siAFP-loaded DDAB-cLNPs (siAFP@DDAB-cLNPs). The results indicated that the relative expression of *AFP* mRNA in HepG2 cells treated with siAFP@DDAB-cLNPs decreased to 77.33 ± 6.16% in comparison to the siSCR@DDAB-cLNPs control group (Figure 5D). Furthermore, our findings demonstrated that both unloaded DDAB-cLNPs and siSCR@DDAB-cLNPs, serving as negative controls, did not induce downregulation of *AFP* mRNA expression, confirming that the reduction of *AFP* mRNA levels is facilitated by siAFP.

### 3.5 Cytotoxicity of siRNA Transfected with LP2000 on Liver Cancer Cells

To ensure that the cause of cell death was not related to excessive transfection of oligonucleotides into the cells, we transfected the siSCR into HepG2 cells using LP2000 for 48 h and measured the viability of the treated cells using the CellTiter-Blue Cell Viability Assay. The results showed that siSCR at a concentration of 100 nM did not impact HepG2 cell viability (Figure 5B). Consequently, we decided to use 100 nM of siSCR@LP2000 as a negative control group in the next study.

Next, to assess the cytotoxic impact of siAFP@LP2000 on HepG2 cell viability, we tested siAFP@LP2000 as well as siSCR@LP2000. The results revealed that HepG2 cells treated with siAFP@LP2000 for 48 h exhibited viability of 47.93 ± 3.83% (Figure 5C). This finding demonstrated that siAFP@LP2000 had the potential to induce noticeable cell death in HepG2 cells. Moreover, siAFP@LP2000 was used as a positive control group for evaluating the cytotoxic effects of siRNA-loaded DDAB-cLNPs on HepG2 cells. According to our optimization, the siRNA at an equivalent concentration of 100 nM was also applied in the DDAB-cLNPs system.

### 3.6 Cytotoxic Studies and Apoptosis Detection in Liver Cancer Cells after Treatment with siRNA-Loaded DDAB-cLNPs

To examine the potential cytotoxic effects of siAFP@DDAB-cLNPs on HepG2 cell viability, we treated HepG2 cells with siAFP@DDAB-cLNPs for 48 h, followed by measuring the decrease in cell viability using the CellTiter-Blue Cell Viability Assay. HepG2 cells treated with siAFP@LP2000 served as the positive control group. The results indicated a reduction in the viability of HepG2 cells treated with siAFP@DDAB-cLNPs to 76.44 ± 3.32%, while the viability of HepG2 cells treated with siAFP@LP2000 decreased to 45.72 ± 3.48% (Figure 5E).

Next, to verify if siAFP@DDAB-cLNPs induce cell death of HepG2 cells via the apoptotic pathway, we measured the activity of caspase-3 and 7 using the Caspase-Glo 3/7 Assay. Typically, active caspase-3 (cleaved caspase-3) is responsible for inducing morphological alterations and DNA fragmentation in cells undergoing apoptosis, while active caspase-7 (cleaved caspase-7) plays a key role in promoting cell detachment from the extracellular matrix during intrinsic apoptosis.^47,48^ In this study, HepG2 cells transfected with siAFP@LP2000 were also used as the positive control group. The findings revealed a significantly elevated activity of caspase-3/7 in HepG2 cells treated with siAFP@DDAB-cLNPs in comparison to both unloaded and siSCR@DDAB-cLNPs control groups. However, the increase in caspase-3/7 activity in HepG2 cells treated with siAFP@DDAB-cLNPs is still not as high as that of the positive control (Figure 5F).

The aforementioned outcomes reveal that the superior anticancer effects of siAFP@LP2000 over our DDAB-cLNPs system are attributed to the composition of LP2000, which includes 2,3-dioleyloxy-*N*-[2-(sperminecarboxamido)ethyl]-*N*,*N*-dimethyl-1-propanaminium (DOSPA), as a cationic lipid, and 1,2-dioleoyl-*sn*-glycero-3-phosphoethanolamine (DOPE), as a neutral helper lipid. This helper lipid facilitates a cationic lipid from endosomal escape at low pH-sensitive conditions, thereby enabling LP2000 to release siRNA more rapidly than the DDAB-cLNPs system.^49,50^ Although the siRNA transportation efficiency of LP2000 in cancer treatment is undeniable, it is still unsuitable for clinical application due to its considerable toxicity.^51^ Thus, our DDAB-cLNPs are an intriguing choice as siRNA carriers because they can be used at higher concentrations and provide anticancer effects that are not much different from the LP2000 system. Moreover, this evidence confirms that our siAFP@DDAB-cLNPs can eliminate liver cancer cells through apoptosis induction, aligning with findings by Chen et al. (2020), who also reported *AFP* expression suppression leading to apoptosis-induced cell death through caspase-3 activation.^9^

## 4 Conclusion

In this study, we have successfully fabricated DDAB-cLNPs as promising siRNA carriers for siAFP delivery into HepG2 cells to treat liver cancer with elevated *AFP* expression. Our nanoparticle platform demonstrated a high capacity for encapsulating siRNA and exhibited efficient internalization into HepG2 cells, resulting in the downregulation of *AFP* mRNA expression. The downregulation of *AFP*, in turn, diminished HepG2 cell viability and led to apoptosis through activation of caspase-3/7. Moving forward, our ongoing study aims to improve the specificity of these nanoparticles to target cells by conjugating the surface of nanoparticles with active ligands specific to liver cancer cells in order to enhance their anticancer efficacy.
